# Producing Insulin from Neural Cells

**DOI:** 10.1371/journal.pmed.0020115

**Published:** 2005-04-26

Before insulin was discovered and purified, doctors could only watch as their patients slowly died of type 1 diabetes mellitus. In January 1922, the prognosis was changed dramatically when a teenager with diabetes in a Toronto hospital became the first recorded recipient of an injection of insulin. Although this preparation was far from perfect, as Frederick Banting said in his Nobel lecture of 1925 (Nobel laureates did not have to wait so long as they do now for recognition), “There was a marked reduction in blood sugar and the urine was rendered sugar free.” Suddenly diabetes was a potentially curable disease. But insulin's very success brought trouble, as demand far exceeded the supply.

Now recombinant insulin is available in plentiful supply, but the control of type 1 diabetes remains far from perfect, and researchers have looked towards an ideal of transplanting insulin-producing cells instead. The best protocol for this, also pioneered in Canada, the Edmonton protocol, uses ß-cells isolated from human cadavers and has shown some remarkable results with around 84% of patients remaining insulin-free after one year and 89% of patients still producing insulin after three years. However, the isolation of ß-cells is laborious and limited by donor availability.[Fig pmed-0020115-g001]

**Figure pmed-0020115-g001:**
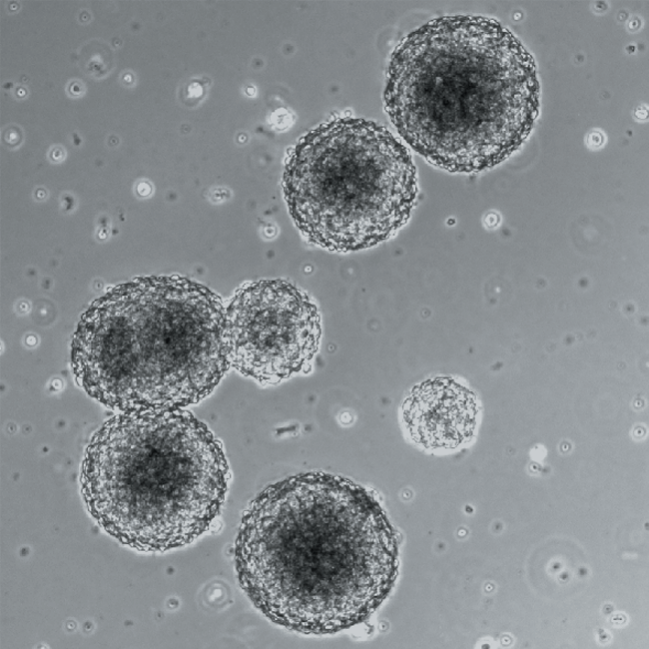
Insulin-producing neurospheres

The next logical step, then, is to look for renewable sources of insulin-producing cells. The emerging science of human stem cell research makes this step possible, and in a paper in this month's *PLoS Medicine* Seung Kim and colleagues from Stanford University suggest that researchers should look beyond just pancreatic precursors and embryonic stem cells to other cell-type precursors for ideas about ß-cell replacement.

The rationale for the approach in this study comes from observations that although the pancreatic islet cells are the principal source of insulin in humans, in some invertebrate species, such as Drosophila, most circulating insulin is produced by brain neurons. Intriguingly, the gene encoding insulin is also transcribed by some vertebrate neurons—although it is not clear whether they then produce or secrete insulin protein.

Kim and colleagues took human neural progenitor cells derived from brain, and exposed them to a series of signals that are known to drive pancreatic islet development. They were able to produce clusters of insulin-producing cells that were responsive to glucose in vitro. After transplantation into immunocompromised mice, circulating human insulin and C-peptide derived from the proinsulin precursor were detected when the mice were given glucose.

Of course, results in mice do not mean that such treatments would automatically work in humans, and before any such therapies become available there are many hurdles to overcome. Some of the most important include the long-term stability and safety of the cells (although the cells remained differentiated in the mice and did not form tumors, such a risk would need to be very thoroughly investigated because of the chronic nature of diabetes), and how to scale up such a process to produce the much larger numbers of cells needed for human treatment. Nonetheless, work like this study from Kim's group might point to where the future of diabetes treatment lies.

